# Identification and validation of a fatty acid metabolism-related lncRNA signature as a predictor for prognosis and immunotherapy in patients with liver cancer

**DOI:** 10.1186/s12885-022-10122-4

**Published:** 2022-10-04

**Authors:** Erbao Chen, Jing Yi, Jing Jiang, Zhilin Zou, Yuqian Mo, Qingqi Ren, Zewei Lin, Yi Lu, Jian Zhang, Jikui Liu

**Affiliations:** 1 School of Medicine, Southern University of Science and Technology, Shenzhen, 518055 Guangdong China; 2 Hepato-Pancreato-Biliary Surgery, Peking University Shenzhen Hospital, Shenzhen, 518036 Guangdong China; 3 Department of Pathology, Peking University Shenzhen Hospital, Shenzhen, 518036 Guangdong China; 4 Department of Ophthalmology, Affiliated Eye Hospital of Wenzhou Medical University, Wenzhou, 325027 Zhejiang China; 5 School of Public Health, Guangdong Medical University, Zhanjiang, 524023 Guangdong China; 6 Guangdong Provincial Key Laboratory of Cell Microenvironment and Disease Research, Shenzhen, 518055 Guangdong China

**Keywords:** Fatty acid metabolism, LncRNA, Immune infiltrations, Hepatocellular carcinoma, Ferroptosis

## Abstract

**Background:**

Fatty acid (FA) metabolism is considered the emerging cause of tumor development and metastasis, driving poor prognosis. Long non-coding RNAs (lncRNAs) are closely related to cancer progression and play important roles in FA metabolism. Thus, the discovery of FA metabolism-related lncRNA signatures to predict outcome and immunotherapy response is critical in improving the survival of patients with hepatocellular carcinoma (HCC).

**Methods:**

FA metabolism scores and a FA metabolism-related lncRNA signature were constructed using a single-sample gene set enrichment analysis based on The Cancer Genome Atlas (TCGA) and Gene Expression Omnibus (GEO) databases. “ConsensusClusterPlus” was used to screen molecular subtypes. Chi-squared test and Fisher’s exact test were applied to explore the relationship between clinical, genomic mutation characteristics and subtypes. Transcription factor (TF) activity scores, cellular distributions, immune cell infiltration, and immunotherapy response were employed to investigate the functions of FA metabolism-related lncRNA signatures. FA metabolism microarray and western blot were performed to detect the biological function of candidate lncRNAs.

**Results:**

A total of 70 lncRNAs that highly correlated with FA metabolism scores in two cohorts were used to construct two distinct clusters. Patients in cluster 2 had lower FA metabolism scores and worse survival than those in cluster 1. Patients in cluster 2 exhibited a high frequency of DNA damage, gene mutations, oncogenic signaling such as epithelial-to-mesenchymal transition, and a high degree of immune cell infiltration. Moreover, the lncRNA signature could predict the effects of immunotherapy in patients with HCC. Furthermore, three lncRNAs (SNHG1, LINC00261, and SNHG7) were identified that were highly correlated with FA metabolism. Additionally, SNHG1 and SNHG7 were found to regulate various FA metabolism-related genes and ferroptosis-related genes in vitro experiments. GSEA analysis revealed that SNHG1 and SNHG7 promote fatty acid beta-oxidation. SNHG1 and SNHG7 silencing dramatically reduced lipid droplets in HCC cells. Many immune-infiltration genes and TFs were overexpressed in HCC tissues with SNHG1 and SNHG7 high expression.

**Conclusions:**

A novel molecular model of FA metabolism-related lncRNAs was developed, which has significantly prognostic potential in HCC diagnosis and aids in clinical decision making.

**Supplementary Information:**

The online version contains supplementary material available at 10.1186/s12885-022-10122-4.

## Background

Hepatocellular carcinoma (HCC) ranks sixth in the global incidence of all tumors. Moreover, the mortality of HCC ranks fourth, accounting for 8.2% of all cancer-related deaths [1]. Although many therapeutic strategies, such as surgical resection, liver transplantation, systemic or local radiotherapy, chemotherapy and targeted immunotherapy are currently used in the treatment of patients with HCC, the median survival time for most advanced patients with HCC is poor [2]. Therefore, discovering new prognostic biomarkers and elucidating novel molecular mechanisms are crucial to improving the treatment modalities of this deadly malignancy.

Metabolic reprogramming, which can promote rapid cancer cell proliferation, invasiveness, metastasis and drug resistance, has become a hallmark of cancer [3, 4]. Cancer cells are also characterized “Warburg effect” [5, 6]. In addition to the abnormity of glucose metabolism, the dysregulation of fatty acid (FA) metabolism has been increasingly recognized as a feature of metabolic reprogramming in cancer [7], especially in HCC [8]. During oncogenesis, FAs synthesize cellular membranes, and produce signaling molecules. Furthermore, studies have indicated that alterations in FA metabolism are involved in HCC progression [9]. Exacerbated de novo lipogenesis is also considered a major metabolic phenotype during HCC initiation and development [10]. However, the biological mechanism underlying FA metabolism in HCC remains unexplored. Thus, the development of a FA metabolism-related genes model could provide a novel approach to HCC therapy.

Long non-coding RNAs (lncRNAs) are RNAs longer than two hundred nucleotides with little ability to encode proteins. Many lncRNAs are widespread in multiple cancers, mediating tumorigenesis and cancer development. They also serve as biomarkers of HCC diagnosis and prognosis. Various studies report that lncRNAs play a critical role in tumor immune regulation and FA metabolism [11, 12]. Linc-Pint inhibited lipogenesis by targeting SRPK2 in the Hepatitis C virus-related liver pathogenesis [13]. LncRNA-NEAT1 mediated abnormal lipolysis via ATGL, driving HCC growth [14]. LncRNA-HR1 inhibited the activity of the SREBP1c promoter and subsequently the expression of FASN, reducing lipid metabolism [15]. However, because most studies only focus on the function of a single lncRNA in HCC FA metabolism, the complete picture of FA metabolism-related lncRNAs in HCC remains unclear.

In the present study, FA metabolism-related genes were downloaded from the MSigDB database. The single sample gene set variation analysis (GSVA) enrichment of FA metabolism was calculated to obtain the FA score of each patient. Pearson correlation coefficient was utilized to identify FA metabolism-related lncRNAs. Additionally, the “ConsensusClusterPlus” analysis was performed to generate FA metabolism-related lncRNA clusters. Subsequently, the clinical characteristics, genomic mutation profiles, signal transduction pathways, immune features, and immunotherapy response between high and low risk groups were compared. A first-order partial correlation analysis revealed Small Nucleolar RNA Host Gene 1 (SNHG1), Long Intergenic Non-Protein Coding RNA 261 (LINC00261), and Small Nucleolar RNA Host Gene 7 (SNHG7) were identified that were highly correlated with FA metabolism. Finally, FA metabolism arrays were used to explore the downstream targets of SNHG1 and SNHG7. The association between SNHG1/7 and ferroptosis was investigated using the the western blot of ferroptosis related-biomarkers.

## Materials and methods

### Data collection

The RNA-sequencing (RNA-seq) data and clinical information on liver hepatocellular carcinoma (LIHC) were downloaded from The Cancer Genome Atlas (TCGA, https://gdc.cancer.gov/). Additionally, the transcriptional expression data and clinical information of the GSE76427 dataset were downloaded from the Gene Expression Omnibus (GEO) database (https://www.ncbi.nlm.nih.gov/geo/). The detailed clinical features are presented in Supplementary excel 1.

### The source of fatty acid metabolism-related genes

The list of FA metabolism-related genes (refer to the genes included in the FA metabolism pathway) was collected from the "HALLMARK FATTY ACID METABOLISM" in the publicly available MSigDB database [16].

### Data pre-processing

The transcriptional data of TCGA were pre-processed as follows: (1) The samples without survival information were excluded; (2) The ENSG were converted into the Gene Symbol. The GSE76427 dataset was pre-processed as follows: (1) The standardized data set was downloaded; (2) The survival time and survival state of the sample were retained; (3) Only the liver cancer samples were obtained; (4) The gene on the chip platform GPL10558 was re-annotated, and the probe was converted into the gene symbol.

### Acquisition of the lncRNA expression profiles

The V32 version of the GTF file was downloaded from the GENCODE website (https://www.gencodegenes.org/), and TCGA expression profiles and GSE76427 datasets were divided into mRNA and lncRNA according to the comments in the file.

### Identification of the FA metabolism-related lncRNAs

The lncRNA expression profiles were annotated from GENCODE (https://www.gencodegenes.org/). Then FA metabolism score of each HCC sample in the TCGA and GSE76427 dataset was calculated using the "GSVA" R package. Single-sample gene set enrichment analysis (ssGSEA) was used to generate the Pearson correlation coefficient and *P* value. The cases were stratified into two subgroups according to the median cut-off of the GSVA enrichment score. A total of 2981 lncRNAs and 182lncRNAs involved in FA metabolism were screened from TCGA LIHC and GSE76427 datasets, respectively, according to the following criteria: correlation |Cor|> 0.25 and *P* < 0.05. The correlation analysis between FA scores and lncRNAs is detailed in Supplementary excel 2 and 3.

### Identification of FA-associated lncRNA subtypes

lncRNAs that were positively or negatively correlated with the FA scores in TCGA and GSE76427 datasets were used for subsequent analysis. The consistency matrix was constructed using consistency clustering (ConsensusClusterPlus), and the samples were clustered and typed [17]. Using the selected FA metabolism score-related lncRNAs, the FA metabolism-related lncRNA subtypes of the sample were obtained. Kaplan–Meier algorithm and Euclidean lows were used to measure distance, and 500 times bootstraps were conducted, each bootstrap process included 80% of the training set patients. The number of clusters was set from 2 to 10, and the best classification was determined according to the consistency matrix and consistency cumulative distribution.

### Gene set enrichment analysis (GSEA) and functional annotation

The "GSEA" package was used to analyze the molecular pathways of different molecular clusters in various biological processes. The candidate gene sets in the Hallmark database were used for GSEA [18]. “ClusterProfiler” package [19] was utilized for functional enrichment annotations.

### Assessment of the TF activity

The activity of TFs was assessed using the method developed by Garcia-Alonso [20] and the level of TF activation among different clusters was compared using analysis of variance (ANOVA).

### A first-order partial correlation analysis

A first-order partial correlation analysis was performed to determine correlations among FA metabolism-related lncRNAs, FA metabolism scores, and FA metabolism-related genes. FA score was postulated x and FA metabolism-related genes levels as y. The first-order partial correlation between x and y under the lncRNA condition was follows:

$${{}_{r_{xylncRNA}=\frac{r_{xy}-r_{xlncRNA}\ast r_{ylncRNA}}{\sqrt{\left(1-r_{x\ln cRNA}^2\right)\ast\left(1-r_{y\ln cRNA}^2\right)}}}}_{}$$

### Risk model

The risk score for each patient was calculated as follows: Score = (beta i × Exp i), where i refers to the expression level of FA metabolism-related lncRNA, and beta refer to the coefficient of the gene regressed by the corresponding lncRNA univariate Cox. The patients were divided into high-risk and low-risk groups according to the optimal segmentation point. The survival curve was drawn using the Kaplan–Meier method for survival analysis, and the logarithmic rank test was performed to assess the significance of the difference.

### Cell lines, cell transfection, RNA isolation, real-Time Quantitative Reverse Transcription- Polymerase Chain Reaction (qRT-PCR), western blot, Oil Red O Staining and statistical analyses

HepG2, Huh7, PLC/PRF/5 and Hep3B cell lines were purchased from the Guangzhou Cellcook Biotech Co.,Ltd (Guangdong, China). Human LO2, HCCL97L, HCCM97H, and HCCLM3 were gifted from Prof. Zhou Zhengjun, Fudan University, Zhongshan Hospital. Small interfering RNA of SNHG7, scramble siRNA of SNHG7, smart silencer of SNHG1 and scramble of SNHG1 were purchased from RiboBio (Guangzhou, China). RNA isolation, qRT-PCR, western blot analysis and statistical analyses were performed as previously described [21]. The primers, siRNA and smart silencer, and antibodies used are listed in Supplementary Table S1, S2 and S3, respectively. The results of qRT-PCR and Immuno-blotting were normalized with β-actin. Oil Red O Staining were performed according to the instructions. The HCC cells were washed twice with PBS, and fixed the cells with 4% paraformaldehyde for 10 min at room temperature and stained the cells with the Oil Red O Kit.

### Ethics statement

Our research protocol was approved by the Ethics Committee of the Peking University Shenzhen Hospital. 36 HCC tissues for validation were obtained from our center. All patients had given permission for their samples to be used in research and the samples were administered by the Peking University Shenzhen Hospital.

## Results

### Construction of a prognostic model based on FA acid metabolism-related lncRNAs

**Fig. 1:**
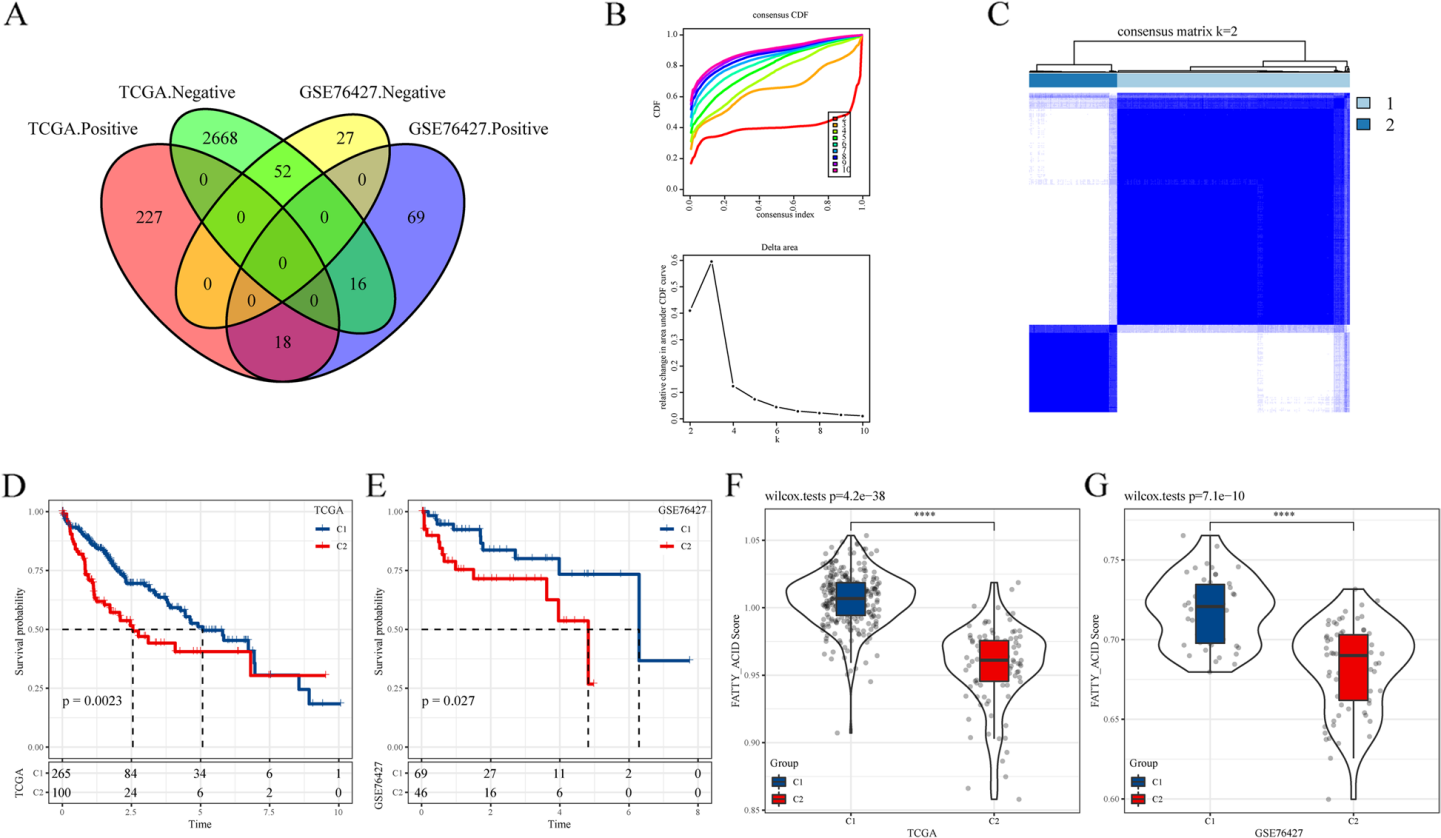
Identifications of FA metabolism related-lncRNA subtypes and their prognostic value in HCC progression. **A** Venn diagram shows that 52 and 18 lncRNAs were negatively and positively correlated, respectively, with FA metabolism in the TCGA and GSE76427 cohorts. **B** The consensus CDF, relative changes in the area under the CDF curves, and tracking plots showed with the index from 2 to 9. **C** The distribution of different clusters with index k = 2. **D-E** Kaplan–Meier plots indicate that the OS of patients in cluster 1 (C1) was significantly longer that those in cluster 2 (C2) in the TCGA. **D** and GSE76427 (**E**) cohorts. **F-G** The FA metabolism scores of C1 were remarkably higher than that of C2 in the TCGA (**F**) and GSE76427 (**G**) cohorts. Abbreviation: FA, Fatty acid; lncRNA, Long non-coding RNA; HCC, Hepatocellular carcinoma; TCGA, The Cancer Genome Atlas; CDF, Cumulative distribution function; OS, Overall survival

To construct FA metabolism-related lncRNA signatures in HCC, 158 genes were extracted from the Hallmark FA metabolism pathway and a single sample GESA were performed to generate an FA metabolism score for each patient. Totally, 2981 and 182 lncRNAs from TCGA LIHC and GSE76427 datasets, respectively, were identified to be significantly related to FA metabolism scores (|Cor|> 0.25 and *P* < 0.05). However, only 70 lncRNAs from the two datasets overlapped and exhibited a co-positve or co-negative correlation with FA metabolism scores (Fig. 1A). Then, “ConsensusClusterPlus”, an unsupervised machine learning algorithm, was used to explore optimal molecular clusters. Two clusters were observed to be the most stable using the Cumulative distribution function Delta area curve (Fig. 1B, C). The overall survival rate of cluster 1 (C1) was significantly longer than that of cluster 2 (C2) (Fig. 1D). Additionally, the same phenomenon was observed in the GSE76427 cohort (Fig. 1E). Furthermore, the FA metabolism scores in C1 were significantly higher than those in C2 in the two cohorts (Fig. 1F, G).

### Relationship between FA metabolism related-lncRNA signatures and clinical characteristics

**Fig. 2:**
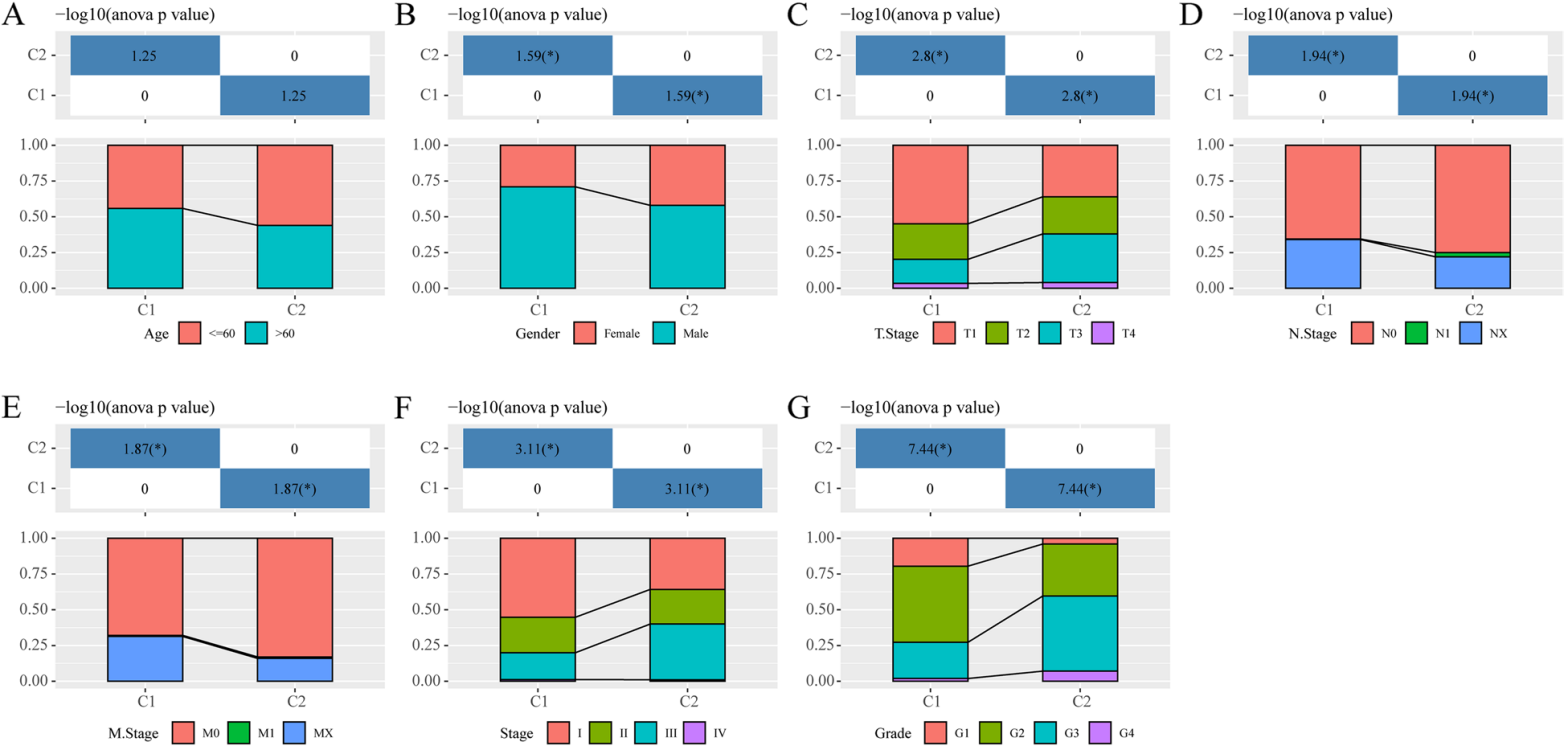
Clinical information distribution of molecular clusters in the TCGA cohort. **A** Age, **B** Gender, **C** T stage, **D** N stage, **E** M stage, **F** Tumor stage, **G** Grade. The table in the upper half represents the chi-square test of the clinical information between different molecular subtypes. Abbreviations: TCGA, The Cancer Genome Atlas

The different clinical features between the two molecular subtypes, which were classified using the FA metabolism related-lncRNA signatures, were investigated. No significant difference was observed in age between the two clusters in the TCGA cohort (Fig. 2A). In line with the conventional speculations that males are more likely to develop liver cancer, the patients in C1 were observed to have a higher percentage of males (Fig. 2B). However, other clinical features, including T stage (tumor stage), N stage (lymph node stage), M stage (tumor metastasis stage), and Grade stage (pathological grade), were showed significant difference between the molecular C1 and C2 (Fig. 2C-G). The patients in C2 were associated with high-grade clinicopathological features, which partly explains the poor prognosis of patients in C2.

### Genomic DNA damage and mutational characterization of high- and low-risk clusters

**Fig. 3:**
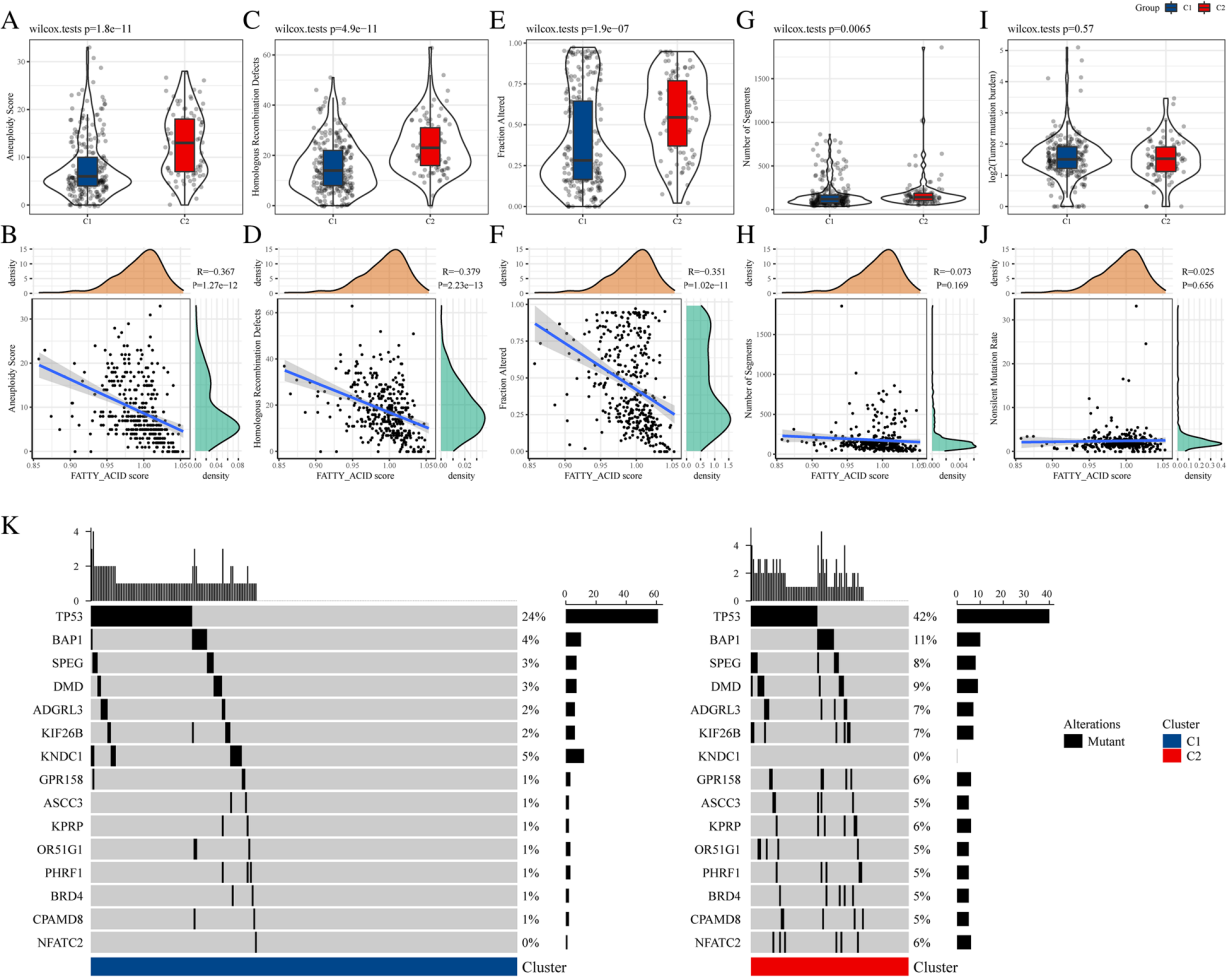
Genomic mutations of the TCGA cohort molecular subtypes. **A**, **C**,** E**,** G**,** I** Comparison of aneuploidy scores, homologous recombination defects, fraction altered, number of segments and nonsilent mutation rates in the molecular subtypes of the TCGA cohort. **B**,** D**,** F**,** H**,** J** Correlation analysis between FA metabolism activity and aneuploidy scores, homologous recombination defects, fraction altered, number of segments and nonsilent mutation rates in the TCGA cohort. **K** Somatic mutation analysis of the two molecular subtypes. Abbreviations: FA, Fatty acid; TCGA, The Cancer Genome Atlas

Malignancy and immune infiltration are associated with DNA damage assessment, including aneuploidy, homologous recombination deficiency (HRD), fraction of altered and number of segments. Aneuploidy is ubiquitously observed in various cancer, reflecting the degree of immune evasion and lower response to immunotherapy [22]. The aneuploidy scores of C1 were significantly lower than that of C2. Moreover, a strong negative correlation between aneuploidy scores and FA metabolism scores were observed in the TCGA cohort (Fig. 3A, B). Additionally, the degree of HRD was negatively correlated with the scores of FA metabolism. The patients in C1 had lesser HRD compare with those in C2. Previous studies reported that apoptosis-resistant tumors are correlated with a higher degree of HRDs and fraction altered but not with mutation rates [23]. Similarly, the level of fraction altered was significantly negatively correlated with the FA metabolism score, and the patients in C1 had lesser fraction altered than those in C2 (Fig. 3C-F). The patients in C1 had a lower number of segments than those in C2 (Fig. 3G). The number of segments were negatively correlated with the FA metabolism score without statistical significance (Fig. 3H). Moreover, the difference in the nonsilent mutation rate between C1 and C2 was not statistically significant (Fig. 3I, J). The mutation burden of C1 was smaller than that of C2, especially, in *TP53, BAP1*, *SPEG*, and *ADRGL3* (Fig. 3K). The patients in C2 had a higher mutation rate of *TP53* (42% vs. 24%), *BAP1* (11% vs. 4%), *SPEG* (8% vs. 3%), and *ADRGL3* (9% vs. 3%), the lower mutation rate of *ATP10D*, *MYO1B*, and *RNF17* (0% vs. 4%) than those in C1 (Supplementary excel 4). Thus, C2 exhibited a higher degree of malignancy.

### Distinct pathway patterns between FA metabolism-related lncRNA subgroups

**Fig. 4:**
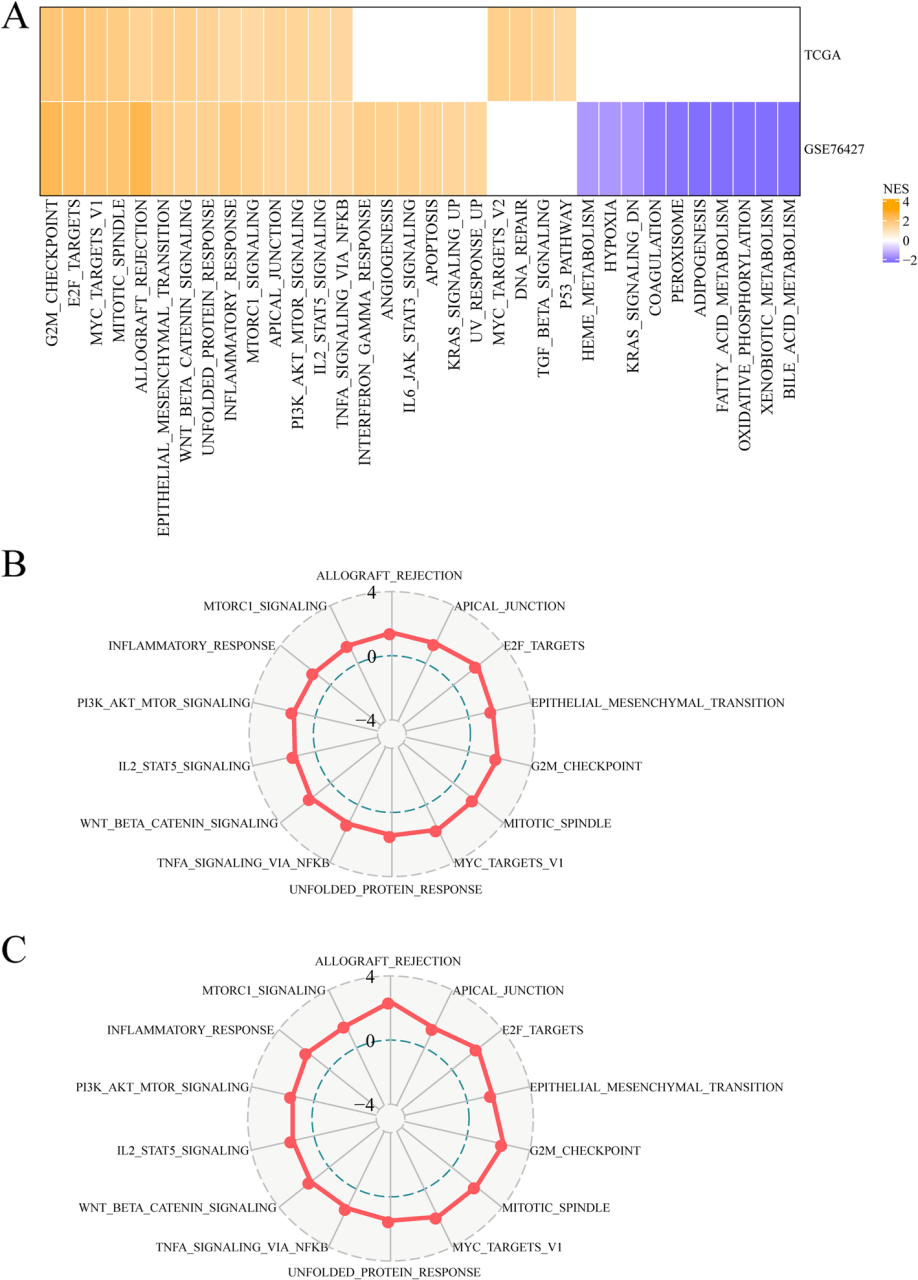
Pathway analysis of the FA metabolism-related lncRNA subgroups. **A** A heatmap demonstrating NESs of the Hallmark pathways calculated by comparing C2 with C1. **B, C** Radar plots indicating the NESs of the Hallmark pathways calculated using GSEA of C2 versus C1 in the TCGA cohort (**B**) and GSE76427 cohort (**C**). Abbreviations: FA, Fatty acid; lncRNA, Long non-coding RNA; NES, Normalized enrichment scores; GSEA, Gene set enrichment analysis

To investigate the activated cellular signaling pathways underlying FA metabolism-associated lncRNAs, a different expressed genes analysis and GSEA between the two clusters were performed. As shown in Fig. 4A, compared with C1, 18 pathways were activated in C2, while 20 pathways were activated and 10 pathways were inhibited in the GSE76427 dataset. In total, 14 oncogenic pathways, including G2M checkpoint, E2F targets, MYC targets, mitotic spindle, allograft rejection, epithelial mesenchymal transition, WNT β-Catenin signaling, unfolded protein response, inflammatory response, mTORC1 signaling, apical junction, PI3K AKT mTOR signaling, IL2 STAT5 signaling, and TNFA signaling via NFKB overlapped in the two datasets (Fig. 4A). Moreover, Radar plots were used to describe consistently up-regulated pathways between C1 and C2 in the two cohorts. Furthermore, GSEA revealed that patients with subtype C2 showed up regulation in EMT, indicating that those lncRNAs could play an important role in connecting the hair surface of EMT, WNT, and tumor necrosis factor (Fig. 4B, C). Thus, the patients in C2 were speculated to possess high level EMT features and immune regulatory-related pathways, indicating that these lncRNAs could play important roles in FA metabolism, EMT, and the immune microenvironment.

### Calculation of immune cell infiltration scores

**Fig. 5:**
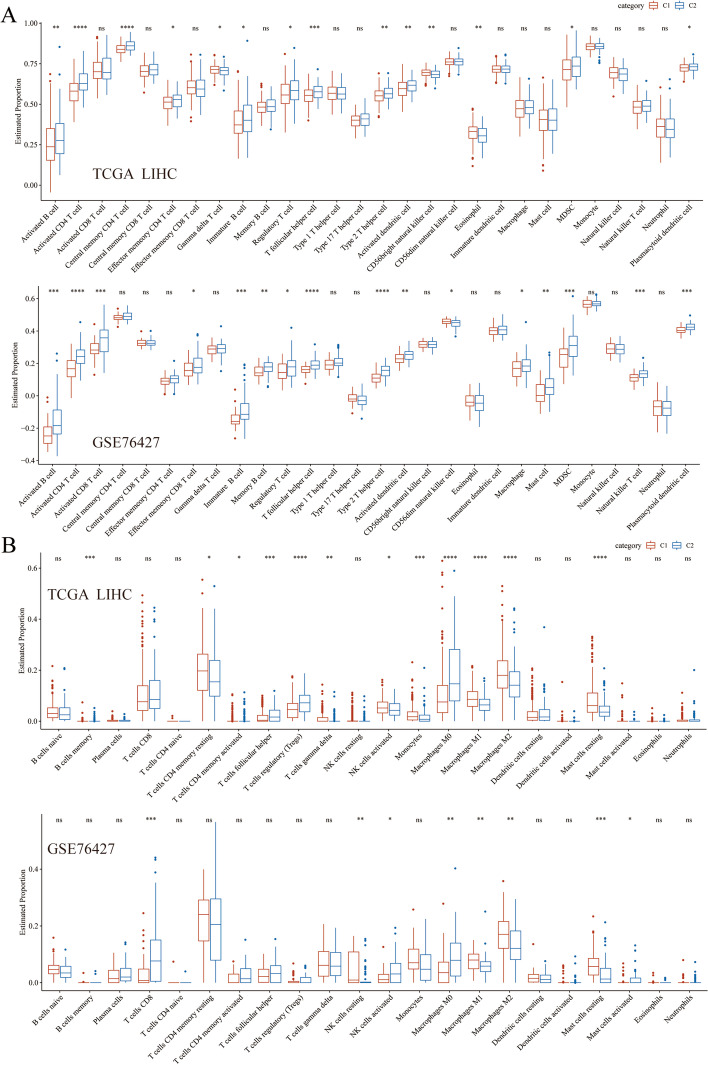
Immune characteristics of FA metabolism-associated lncRNA subsets. **A** Differences in 28 immune infiltration scores were assessed using ssGSEA in the TCGA and GSE76427 cohorts. **B** The difference in 22 immune infiltration scores were evaluated using CIBERSORT in the TCGA and GSE76427 cohorts. Abbreviations: FA, Fatty acid; lncRNA, Long non-coding RNA; ssGSEA, single sample gene set enrichment analysis; TCGA, The Cancer Genome Atlas

The infiltration of immune cells, according to RNA-sequencing, was determined based on a previous study [24]. As shown in Fig. 5A, activated B cell, activated CD4 T cell, immature B cell, regulatory T cell, T follicular helper cell, type 2 T helper cell, activated dendritic cell, MDSC, and plasmacytoid dendritic cell in C2 were more significant than those in C1 in the TCGA LIHC and GSE76427 cohorts (Fig. 5). Additionally, CIBERSORT was used to assess the degree of immune cell infiltration in the TCGA LIHC and GSE76427 cohorts. The differences between C1 and C2 were mainly concentrated in the number of macrophages. The M0 macrophages were significantly upregulated in C2 in TCGA LIHC and GSE76427, while M1 and M2 macrophage were significantly downregulated in C2. Besides macrophages, mast cells resting were significantly decreased in C2 compared to C1. The proportion of activated NK cells was decreased in the TCGA LIHC cohort. However, activated NK cells in C2 were higher than that in C1 in the GSE76427 cohort (Fig. 5B).

### Predictive value of the immunotherapy response of FA metabolism-related lncRNA signatures

**Fig. 6:**
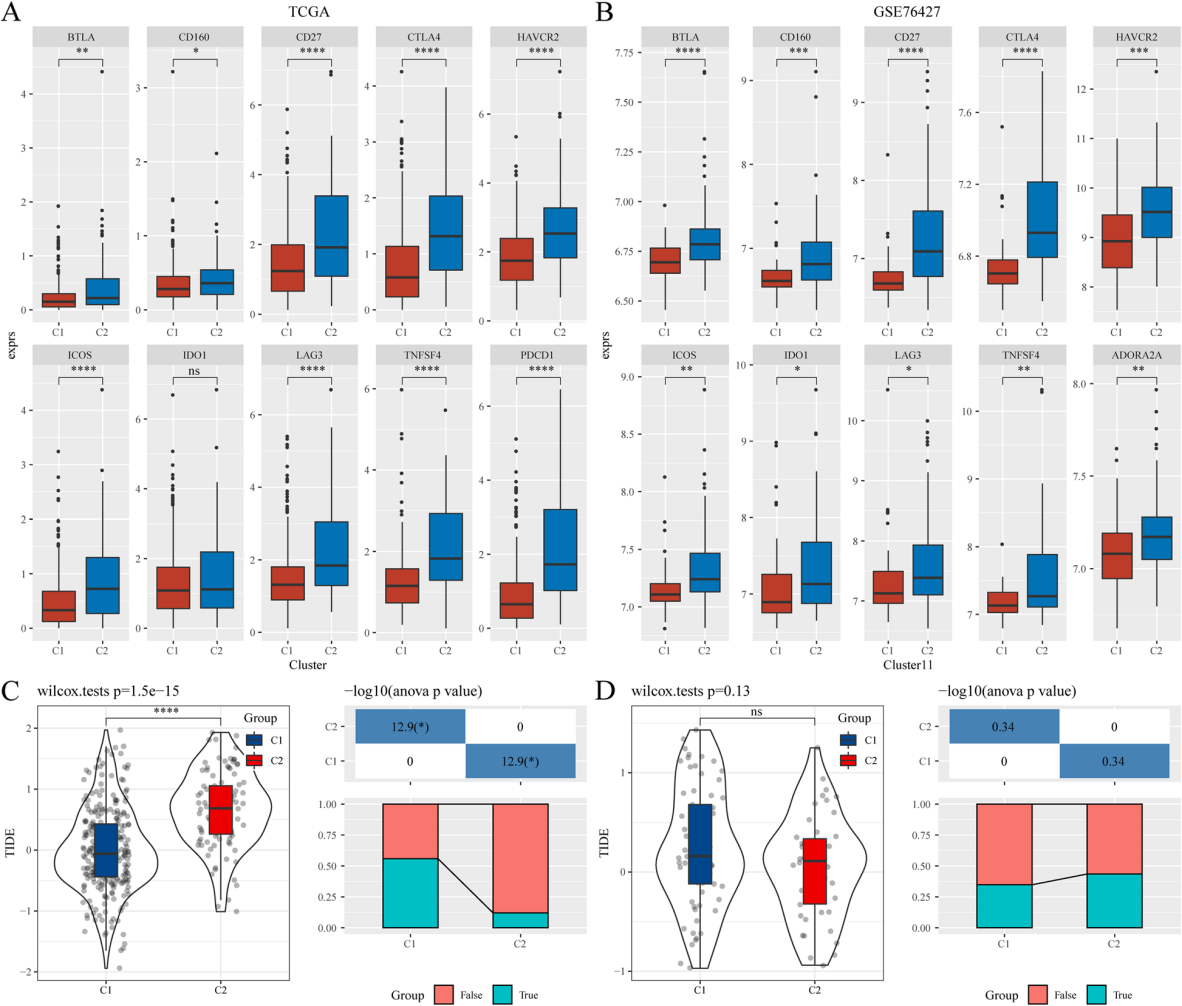
Analysis of the difference in immunotherapy in FA metabolism-related lncRNA subgroups. **A**,** B** The boxplots display the immune checkpoints that were up-regulated in C1 compared with C2 in the TCGA and GSE76427 cohorts. **C** Differences in the TIDE score and immune response status of the different molecular subtypes in the TCGA cohort. **D** Differences in the TIDE score and immune response status of the different molecular subtypes in the GSE76427 cohort. Abbreviations: FA, Fatty acid; lncRNA, Long non-coding RNA; TCGA, The Cancer Genome Atlas; TIDE, Tumor Immune dysfunction and exclusion

Given the importance of immunotherapy in HCC treatment, the immune checkpoint genes in the HisgAtlas dataset were analysed [25], revealing that many immune checkpoint genes were significantly highly increased in C2 than that in C1. As shown in Fig. 6A, B and Supplementary Fig. 2, the high-risk subgroups of C2 had overexpression of several critical immune checkpoint genes, such as BTLA, CD160, CD27, CTLA4, HAVCR2(TIM-3), ICOS, IDO1, LAG3, TNFSF4, PDCD1 (PD-1), and ADORA2A, compared to those of C1. Additionally, tumor Immune dysfunction and exclusion (TIDE) software was used to assess the potential response to immunotherapy in the two clusters. The higher the TIDE predictive score, the higher the chance of immune escape, indicating that patients are less likely to benefit from immunotherapy (Fig. 6C, D). TIDE scores in C2 were higher than that of C1 in the TCGA cohort, suggesting that patients in C2 have a higher possibility of immune escape and lower benefits from immunotherapy (Fig. 6C). However, no significant difference was observed in the predictive value of immunotherapy response in the GSE76427 cohort (Fig. 6D).

### Characterization of FA metabolism-related lncRNAs

**Fig. 7:**
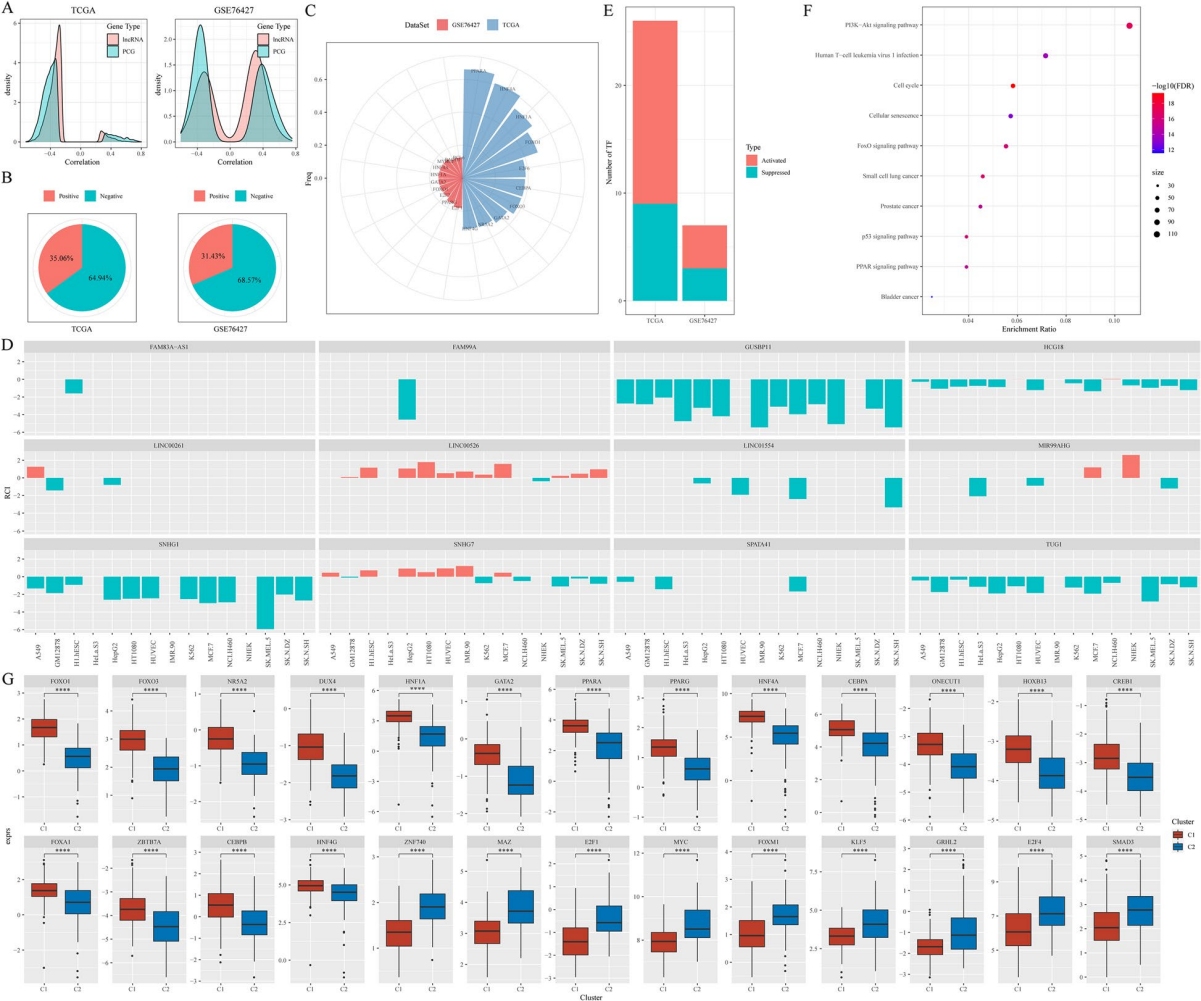
Integrative Analysis of FA metabolism-related lncRNAs. **A** Correlation coefficient density curve of FA metabolism-related lncRNAs and PCG. **B** The cellular component of FA metabolism-associated lncRNAs in cell lines. RCI < 0 indicates nuclear localization and RCI > 0 indicates cytoplasmic localization. **C** The distribution of TFs has a significant negative correlation with nuclear lncRNAs in the two cohorts. **D** Locations of the 12 lncRNAs in the signature. **E** Distribution of the activated and suppressed TFs in C2 compared with C1 in the TCGA and GSE76427 cohorts. **F** The results of the functional enrichment analysis of TFs up-regulated in the C1 subtype. **G** The dysregulation in TF activity of the up-regulated transcription factors in the TCGA subtypes. Abbreviations: FA, Fatty acid; LncRNA, Long non-coding RNA; HCC, Hepatocellular carcinoma; TCGA, The Cancer Genome Atlas; PCG, Protein coding gene; RCI, relative concentration index; TF, Transcriptional factor

Unlike protein-coding genes (PCG), most FA metabolism-related lncRNAs were negatively correlated with FA scores (Fig. 7A). Previous studies have shown that the function of lncRNA partly depends on its cellular localization. Usually, lncRNAs in the nucleus can activate or inhibit the transcriptional activities of target genes by directly binding to them, and regulating gene expression by participating in histone modification or recruitment of TFs [26]. lncRNAs in the cytoplasm usually interact with miRNAs as competitive endogenous RNA and mediate target gene expression [27]. “Relative concentration index (RCI)”, as defined by LncATLAS database [28] was used to describe the proportion of lncRNAs in the cytoplasm and nucleus. Additionally, 64.94% of the RCI was negative in the TCGA cohort, while 68.57% of the RCI was negative in the GSE76427 cohort, suggesting that approximately 70% of FA metabolism-related lncRNAs are localized in the nucleus (Fig. 7B).

Furthermore, the relationship between FA metabolism-related lncRNAs and dysregulated TFs was analyzed. To compare the TF activity between the two clusters, the TF activity score of each sample from TCGA and GSE76427 was calculated using the method developed by Garcia-Alonso [20]. A total of 123 and 70 significantly activated TFs were identified in C2 in the TCGA and GSE76427 cohorts, respectively. Additionally, analyzing correlation between nuclear lncRNAs and differential expressed TFs revealed a significantly negative correlation between a group of FA metabolism-related lncRNAs and a set of TFs (Fig. 7C). A total of 18 lncRNAs were positively correlated with FA scores, while the remaining 52 lncRNAs were negatively correlated with FA metabolism scores. Based on the correlation between lncRNAs and TF activity, as shown in Fig. 7D, most of the 12 lncRNAs in the signature were located in the nucleus. Compare with C1, nine TFs were significantly decreased and 17 TFs were significantly increased in C2 in the TCGA dataset, while four TFs were significantly down-regulated and three TFs were significantly up-regulated in C2 compared with C1 in the GSE76427 dataset (Fig. 7E). These 26 TFs were speculated to contribute to poor prognosis in C2. To validate this hypothesis, a cellular signaling pathway enrichment analysis of the 26 TFs was performed. The results showed that the downstream targets by the TFs were significantly enriched in a few critical cancer-related pathways, such as PI3K-Akt signaling pathway, cell cycle, FoxO signaling pathway, small cell lung cancer, p53 signaling pathway (Fig. 7F, Supplementary excel 5). Furthermore, 17 TFs expression were significantly increased and 9 TFs expression were significantly decreased in C2 compared with C1 in the TCGA dataset (Fig. 7G). Therefore, these FA metabolism-related lncRNAs and TFs could together promote HCC development.

### Identification of key lncRNAs related to FA metabolism

**Fig. 8:**
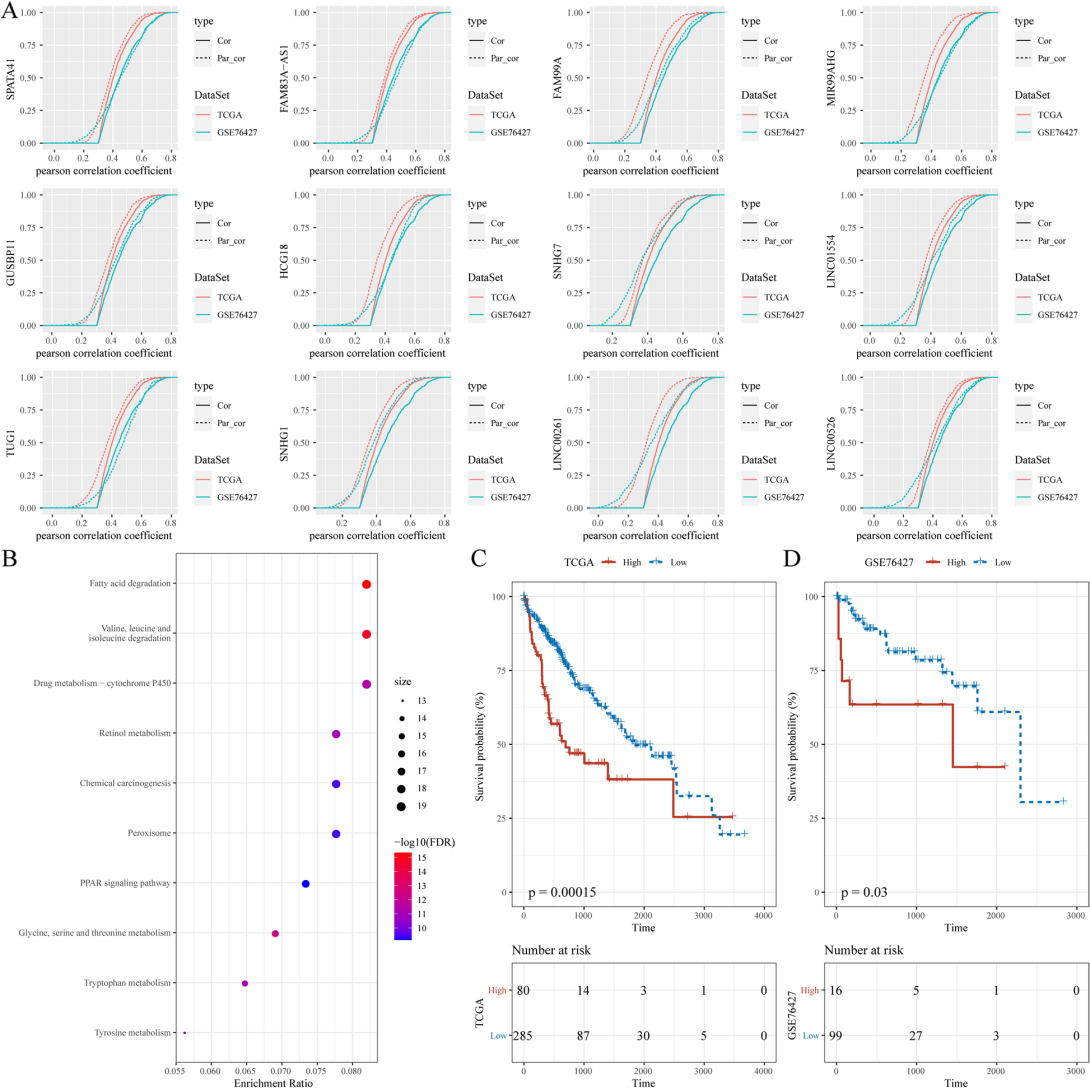
Identification of core lncRNAs related to FA metabolism. **A** The CDF of FA metabolism-related genes, with or without adjusting the first-order partial correlation of lncRNA. The solid line represents the CDFs of the correlation coefficient between the unadjusted FA metabolism scores and gene expression, and the dotted line represents the first-order partial correlation adjustment relationship between FA metabolism scores and gene expression. The two distributions were compared using the Kolmogorov–Smirnov test. The x-axis represents the Pearson’s correlation coefficient between FA metabolism scores and gene expression, and the y-axis represents the cumulative probability. **B** Enrichment analysis of genes significantly related to lncRNAs. **C**,** D** Kaplan–Meier curve of the high- and low- risk clusters in the TCGA and GSE76427 cohorts. Abbreviations: FA, Fatty acid; lncRNA, Long non-coding RNA; CDF, Cumulative distribution curve

To investigate the most crucial lncRNA in FA metabolism regulation, a first-order partial correlation analysis between FA metabolism scores and lncRNAs was performed. When the contribution of three key lncRNAs (SNHG1, LINC00261, and SNHG7) was removed, the correlation between FA metabolism scores and FA metabolism-related protein-coding genes (PCGs) decreased significantly (Fig. 8A), indicting their critical roles in linking FA metabolism and related signaling pathways. Then, using the FA metabolism-related PCGs and three key lncRNAs, consistent FA metabolism-related genes were identified, which were significantly enriched in FA degradation, drug metabolism-cytochrome P450, retinol metabolism, PPAR signaling pathway, and other signaling pathways (Fig. 8B). Base on the expression of the three key lncRNAs, the samples were divided into high- and low-risk groups using the best segmentation point. The overall survival of the high-risk groups was significantly shorter than the low-risk group in the two HCC datasets (Fig. 8C, D). Thus, the three lncRNAs occupy the core position in FA metabolism and are associated with the prognosis of patients with HCC.

### The biological function of SNHG1 and SNHG7 in HCC cells

**Fig. 9:**
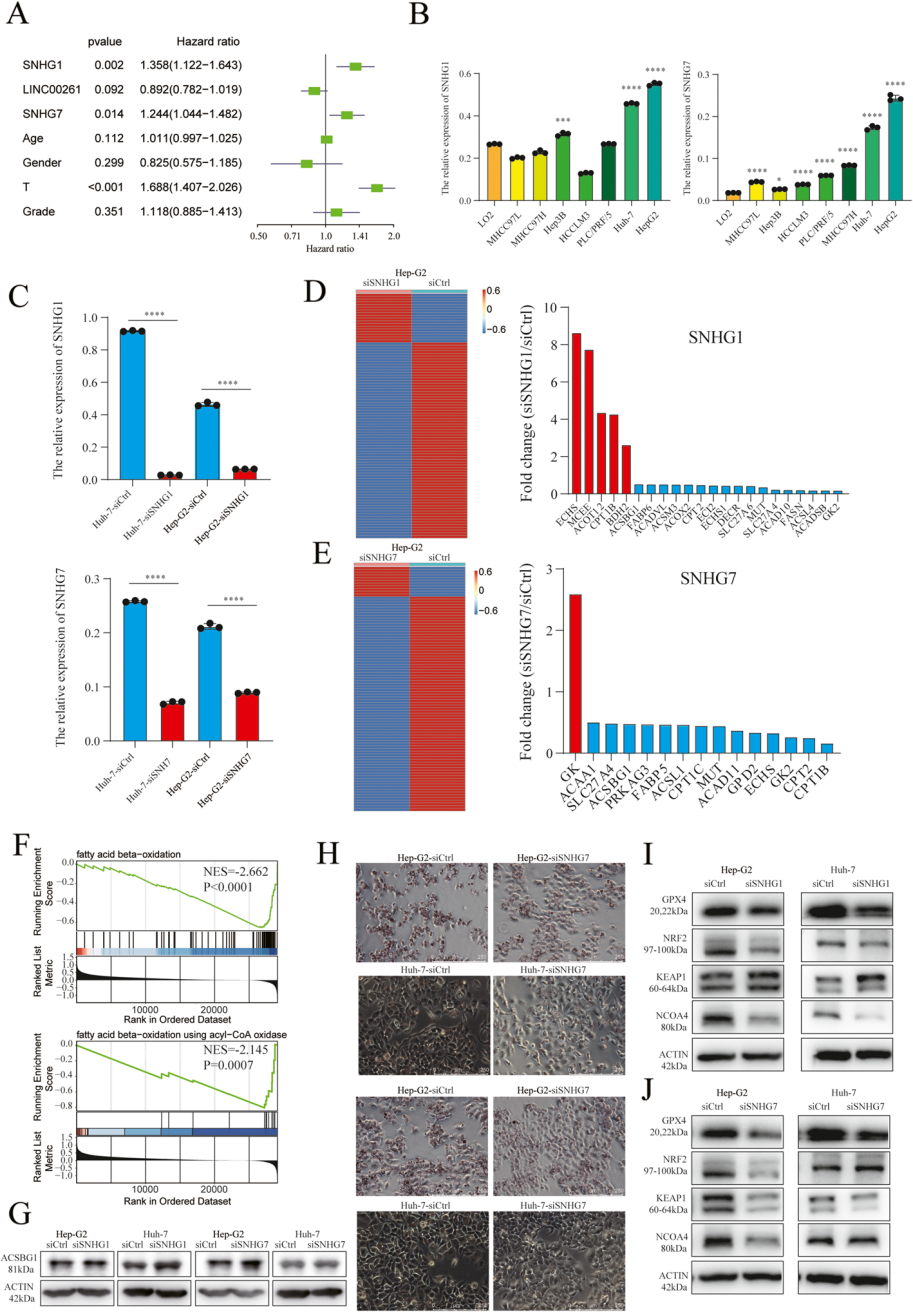
Experimental validation of SNHG1 and SNHG7 in HCC cell lines. **A** Univariate Cox analysis revealed that SNHG1 and SNHG7 were independent prognostic factors for HCC. **B** qRT-PCR analysis showed that the expression of SNHG1 was increased in the hepG2 and Huh7 cell lines, and the expression of SNHG7 was upregulated in most HCC cell lines. **C** Smart silencer-mediated knockdown of SNHG1 and the siRNA-mediated knockdown of SNHG7 were confirmed using qRT-PCR. **D** The results of the FA metabolism PCR array show that the expression of 5 genes (ECHS1, MCEE, ACOT12, CPT1B, and BDH2) were up-regulated and that of 17 genes ACSBG1, FABP6, ACADVL, ACSM3, ACOX2, CPT2, ECI2, ECHS1, DECR, SLC27A6, MUT, SLC27A4, ACAD10, FASN, ACSL4, ACADSB, and GK2 were down-regulated after the knockdown of SNHG1 in the HepG2 (I Fold change I > 2). **E** The results of the FA metabolism PCR array show that the expression of GK was up-regulated, while that of 14 genes (ACAA1, SLC27A4, ACSBG1, PRKAG3, FABP5, ACSL1, CPT1C, MUT, ACAD11, GPD2, ECHS1, GK2, CPT2, and CPT1B) were down-regulated after silencing SNHG7(I Fold change I > 2). **F** GSEA analysis revealed that the signature was negatively regulate the fatty acid beta-oxidation, and fatty acid beta-oxidation using CoA oxidase. **G** ACSBG1 expression was increased after the knockdown of SNHG1 in the HepG2 and Huh7 cell lines, and ACSBG1 expression was increased after the knockdown of SNHG7 in the HepG2 and Huh7 cell lines. **H** The cells were stained with Oil Red O kit and photographed to lipid droplets. **I** GPX4, NRF2, NCOA4, and CD98 expressions were downregulated and that of KEAP1 was upregulated after the knockdown of SNHG1 in the HepG2 and Huh7 cell lines. **J** GPX4, NRF2, NCOA4, and CD98 expressions were downregulated and that of KEAP1 was upregulated after the knockdown of SNHG1 in the HepG2 and Huh7 cell lines. *, *P* < 0.05, ****, *P* < 0.0001. The original blots/gels are presented in Supplementary Fig. 2

To further investigate the molecular function of the three FA metabolism-related lncRNAs, cox analysis was performed, which revealed that the expressions of SNHG1 and SNHG7 were significantly correlated with prognosis (*P* < 0.05), while LINC00261 was correlated with the prognosis without statistically significance (*P* = 0.092) (Fig. 9A). Thus, SNHG1 and SNHG7 were used for subsequent analysis. The results of qRT-PCR showed that the expression of SNHG1 was up-regulated in the hepG2 and Huh7 cell lines, while that of SNHG7 was upregulated in the most HCC cell lines (Fig. 9B). Additionally, lncRNA silencer was used to knock down SNHG1 expression, because SNHG1 is in the nucleus, while siRNA was used to inhibit SNHG7 expression, because SNHG7 is located in the cytoplasm. Smart silencer-mediated knockdown of SNHG1 and siRNA-mediated knockdown of SNHG7 were confirmed using qRT-PCR (Fig. 9C). FA Metabolism PCR Array was applied to explore the potential targets of SNHG1 and SNHG7, wherein, many FA metabolism-related genes were decreased on SNHG1 and SNHG7 knockdown. Among these potential downstream genes, five genes (ECHS1, MCEE, ACOT12, CPT1B, and BDH2) were up-regulated and 17 genes (ACSBG1, FABP6, ACADVL, ACSM3, ACOX2, CPT2, ECI2, ECHS1, DECR, SLC27A6, MUT, SLC27A4, ACAD10, FASN, ACSL4, ACADSB, and GK2) were decreased after SNHG1 knockdown in the HepG2 cell line (I Fold change I > 2) (Fig. 9D). Moreover, GK was up-regulated, and 14 genes (ACAA1, SLC27A4, ACSBG1, PRKAG3, FABP5, ACSL1, CPT1C, MUT, ACAD11, GPD2, ECHS1, GK2, CPT2, and CPT1B) were down-regulated after SNHG7 silencing (I Fold change I > 2) (Fig. 9E). Additionally, the signature negatively regulated fatty acid oxidation and fatty acid beta-oxidation using CoA oxidase among FA metabolism through GSEA analysis (Fig. 9F). ACSBG1 possesses long-chain Acyl-CoA synthetase, which activates fatty acids to their CoA derivatives, plays a central role in fatty acid metabolism. Previous literature revealed that ACSBG1 promote tumors to be more sensitive to ferroptosis. ACSBG1 was identified as a key pro-ferroptotic factor in this heat-induced ferroptosis process [29]. Thus, we chose ACSGB1 to perform western blot analysis. The results of western blot showed that the expression of ACSBG1 were significantly increased after SNHG1 inhibition, while the level of ACSBG1 is slightly increased after SNHG7 inhibition (Fig. 9G). Moreover, we performed the Oil Red O staining experiments and found that the amounts of intracellular lipid droplets are significantly decreased after knockdown of SNHG1 or SNHG7 in HepG2 and Huh 7 cell lines (Fig. 9H). Ferroptosis is recognized as a non-traditional form of programmed cell death, characterized by iron overload and lipid peroxidation. The cross-talk between ferroptosis and FA metabolism plays an important role in cancer progression. Hence, several key proteins were investigated by knocking down SNHG1 and SNHG7 in the HepG2 and Huh 7 cell lines. Moreover, GPX4, NRF2, NCOA4, and CD98 were downregulated whereas KEAP1 was upregulated after the knockdown of SNHG1 in the HepG2 and Huh7 cell lines (Fig. 9I). The levels of GPX4, NRF2, NCOA4, and CD98 were downregulated while that of KEAP1 was upregulated after the knockdown of SNHG7 in the HepG2 and Huh7 cell lines (Fig. 9J). Thus, SNHG1/7 not only play important role in FA metabolism, but also in ferroptosis.

### The validation of SNHG1 and SNHG7 in HCC tissues

**Fig. 10:**
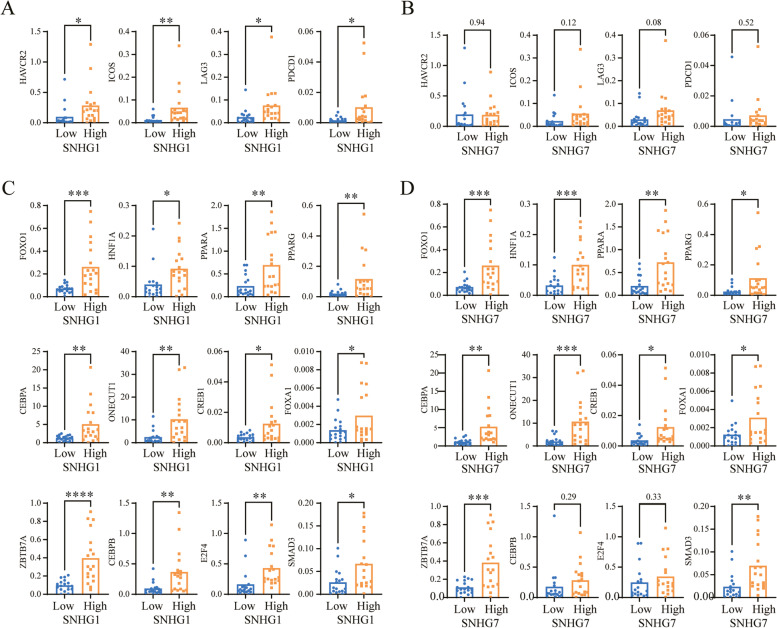
The validation of the signature in clinical HCC tissues**. A** The immune-infiltration related genes (HAVCR2, ICOS, LAG3, and PDCD1) were significantly increased in the SNHG1 high expression group. **B** the level of HAVCR2, ICOS, LAG3, and PDCD1 were slightly increased without significance in the SNHG7 high expression group. **C** The transcription factors (FOXO1, HNF1A, PPARA, PPARG, CEBPA, ONECUT1, CREB1, FOXA1, ZBTB7A, CEBPB, E2F4, and SMAD3) were remarkably overexpressed in the SNGH1 high expression group., the expression of FOXO1, HNF1A, PPARA, PPARG, CEBPA, ONECUT1, CREB1, FOXA1, ZBTB7A, and SMAD3 were significantly overexpressed in the SNGH7 high expression group. *, *P* < 0.05, **, *P* < 0.01. ***, *P* < 0.001, ****, *P* < 0.0001

To validate our findings, we performed qRT-PCR analysis using 36 liver cancer tissue specimens from our center. According to the expression levels of SNHG1 or SNHG7, we divided 36 HCC tissues into high and low expression groups. We observed that the immune-infiltration related genes (HAVCR2, ICOS, LAG3, and PDCD1) were significantly increased in the SNHG1 high expression group (Fig. 10A), however, the level of HAVCR2, ICOS, LAG3, and PDCD1 were slightly increased without significance in the SNHG7 high expression group (Fig. 10B). On the other hand, the transcription factors (FOXO1, HNF1A, PPARA, PPARG, CEBPA, ONECUT1, CREB1, FOXA1, ZBTB7A, CEBPB, E2F4, and SMAD3) were remarkably overexpressed in the SNGH1 high expression group (Fig. 10C), the expression of FOXO1, HNF1A, PPARA, PPARG, CEBPA, ONECUT1, CREB1, FOXA1, ZBTB7A, and SMAD3 were significantly overexpressed in the SNHG7 high expression group (Fig. 10D).

## Discussion

Several studies have shown that FA metabolism, a critical metabolic pathway that, provides energy, and signaling factors promotes malignancy [30, 31]. Moreover, lncRNAs can mediate cancer growth, invasion and metastasis. However, clinical association between lncRNAs and FA metabolism remains unclear. A systematic analysis was performed to identify FA metabolism-associated lnRNAs in HCC, which revealed that FA metabolism-related lncRNA signatures remarkably correlated with the prognosis of HCC in both the TCGA and GEO datasets. Furthermore, on comprehensively analyzing, the C1 and C2 subtypes, significant differences in clinical features, gene mutations, oncogenic pathways, TF activities, and immune status were observed. Finally, three key lncRNAs, namely SNHG1, LINC00261, and SNHG7, were identified, which showed significant correlations with FA metabolism according to the first-order partial correlation analysis. Notably, SNHG1 and SNHG7 not only regulated many FA metabolism related-genes, but also mediated cancer cell ferroptosis.

To evaluate the FA metabolism score of each clinical sample, ssGSEA-derived FA metabolism score was performed instead of directly correlating of FA metabolism-related genes due to the huge number of FA metabolism-related genes. The direct association of lncRNAs with these genes generates hundreds of *P* values, leading to several false-positive results and hindering the assessment of the significance of the association between each lncRNA and FA metabolism. To circumvent this problem, a ssGSEA algorithm was performed to include 158 FA metabolism-related genes as a gene set to produce a score for each patient. The GSVA enrichment score of FA metabolism for individual patients was obtained and followed by the degree of FA activation. Thus, the score of FA metabolism-related lncRNA signatures was significantly associated with the outcome of HCC using two independent datasets.

On comparing clinical information, gene mutation, and immune-features between the two clusters, the patients in C2 had higher possibilities of DNA damage features, such as aneuploidy, HRD, fraction altered, and the number of segments, and higher degree of genomic mutation in C2 with worse prognosis. To a certain extent, these results could explain the poor survival of C2. Additionally, several gene mutations were associated with FA metabolism related-lncRNAs. For example, the mutations of *TP53*, *BAP1*, and *DMD* were the top three gene mutations in C2. Takai et al. reported that patients with HCC patients having the *TP53* mutation are associated with worse clinical tumor stage and prognosis [32]. Previous studies report that the *TP53* mutation suppresses immune response in HCC, with its mechanism associated with higher infiltration of immunosuppressive cells [33]. In the tumor microenvironment, the differences between C1 and C2 were mainly concentrated in the number of macrophages. The M0 macrophages was significantly upregulated in C2 in TCGA LIHC and GSE76427, while M1 and M2 macrophage were significantly downregulated in C2, suggesting strong heterogeneity between C1 and C2, at multiple levels, including clinical, molecular, and immunological levels.

Recently, drugs targeting FA metabolism in T cells have been reported to improve the efficacy of immunotherapy. TVB3664, a FASN inhibitor, combined with PD-L1 antibody could improve therapeutic efficacy [34]. Xiao et al. revealed that lipid peroxidation contributed to Tc9 cell longevity and enhanced T-cell based immunotherapy [35]. The EMT process has also been reported to be associated with cancer cell immune evasion. However, molecular relationships among FA metabolism, EMT and immunoregulation in cancer development remained unexplored. In the present work, various types of immune cell types, immune checkpoint genes and EMT process were enriched in patients in C2, as stratified by FA metabolism-related lncRNAs. These results indicated that the tumor microenvironment of C2 is surrounded by numerous immune cells. Additionally, the EMT process was correlated with the cancer cell immune escape [36–39], suggesting that the EMT activation in C2 stimulates HCC cells to form an immunosuppressive environment, and protecting HCC cells from recognition and attack by immune cells. Based on these differences, the TIDE tool was used to further assess the potential response to immunotherapy. The C2 exhibited higher TIDE scores than C1 in the TCGA cohort, implying that C2 has a higher chance of immune escape. Thus, the patients in C2 are less likely to benefit from immunotherapy. Overall, these findings suggest that the FA metabolism-associated lncRNA model can predict the prognosis and immunotherapy response of patients with HCC.

Finally, in exploring the lncRNAs that strongly regulate FA metabolism using first-order partial correlation analysis, three key lncRNAs, namely SNHG1, LINC00261, and SNHG7, had significant correlations with FA metabolism scores were identified. These three key lncRNAs not only reflect the FA metabolism pattern, but also can predict the prognosis of patients with HCC. Furthermore, SNHG1 and SNHG7 were identified as independent factors for HCC prognosis. Previous studies report that SNHG1 promotes HCC development and metastasis through sponging miR-195-5p [40], miR-377-3p [41], miR-376a [42], and miR-326 [43]. Additionally, SNHG7 contributes to HCC growth, invasion and metastasis via miR-122-5p [44], miR-9-5p [45], and miR-34a [46]. To the best of our knowledge, this is the first to elucidate association between SNHG1/7 and FA metabolism. PCR analysis revealed that many genes, including ACSBG1, CPT2, and GK2, were regulated by SNHG1/7. The level of lipid peroxidation-product accumulation, which is mediated by the producing and scavenging of lipid peroxide, is regarded as determining factors of ferroptosis occurrence [47]. Therefore, the changes in some ferroptosis-related genes were explored, wherein, SNHG1/7 was found to not only regulate many FA metabolism related-genes, but also mediate cancer cell ferroptosis. GPX4, NRF2, NCOA4, and CD98 expressions were downregulated after SNHG1 knockdown, and GPX4, NRF2, NCOA4, and CD98 expressions were downregulated after SNHG7 knockdown, indicating that SNHG1/7 also are involved in ferroptosis in HCC.

## Conclusions

FA metabolism-related lncRNA signatures were developed from two independent HCC cohorts of TCGA and GEO. Furthermore, a cluster of patients with HCC was identified using the lncRNA signature, which had poorer survival, a higher infiltration of immune-suppressive cells, and a lower response to immunotherapy. SNHG1/7 were also revealed to regulate FA metabolism and ferroptosis. Therefore, this study provides novel insights into the clinical aspects of HCC.

## Supplementary Information

**Additional file1: Supplementary excel 1.** Detailed clinical features in the two HCC cohorts.

**Additional file2: Supplementary excel 2.** The correlation analysis between FA scores and lncRNAs in the TCGA LIHC cohort.

**Additional file3: Supplementary excel 3.** The correlation analysis between FA scores and lncRNAs in the GSE76427 cohort.

**Additional file4: Supplementary excel 4.** The mutation profiles in the TCGA LIHC cohort.

**Additional file5: Supplementary excel 5.** The KEGG analysis of TFs in the TCGA LIHC cohort.

**Additional file6: Supplementary figure 1.** The expression of immune checkpoint genes between FA metabolism-related lncRNA subgroups. (A, B) The boxplots display the dysregulation of immune checkpoint genes in the TCGA and GSE76427 cohorts.

**Additional file7: Supplementary figure 2.** Original gels for all western blots in Figure 9 Original gel image measuring immunopositivity against ferroptosis markers in HCC cells. ACTIN was used as loading control. Bands used in the manuscript have been boxed in red.

**Additional file8: Supplementary figure 3.** Original gels images for all western blots in Figure 9.

**Additional file9: Supplementary Table 1.** Sequences of Primers used for qRT-PCR.

**Additional file10: Supplementary Table 2.** Sequences of siRNA and smart silencer used for lncRNA knockdown.

**Additional file11: Supplementary Table 3.** List of Primary Antibodies used in the study.

## Data Availability

The datasets used in the current study are openly available in TCGA (https:// cancergenome.nih.gov/), GEO (https://www.ncbi.nlm.nih.gov/geo/), and MSigDB (https://www.gsea-msigdb.org/gsea/msigdb/). All date and materials are available from the corresponding authors upon request.

## Abbreviations

LncRNA: Long Non-coding RNA
HCC: Hepatocellular Carcinoma
FA: Fatty Acid
TCGA: The Cancer Genome Atlas
GEO: Gene Expression Omnibus
TF: Transcription factor
SNHG1: Small nucleolar RNA host gene 1
LINC00261: Long intergenic Non-protein coding RNA 261
SNHG7: Small nucleolar RNA host gene 7
SRPK2: SRSF protein kinase 2
LncRNA-NEAT1: Nuclear paraspeckle assembly transcript 1
ATGL: Patatin like phospholipase domain containing 2
SREBP1c: Sterol regulatory element binding transcription factor 1
MsigDB: The molecular signatures database
GSVA: Gene set variation analysis
LIHC: Liver hepatocellular carcinoma
GSEA: Gene set enrichment analysis
ANOVA: Analysis of variance
qRT-PCR: Real-Time quantitative reverse transcription- polymerase chain reaction
HRD: Homologous recombination deficiency
TIDE: Tumor Immune Dysfunction and Exclusion
PCG: Protein-coding Genes
RCI: Relative Concentration Index
ACSBG1: Acyl-CoA Synthetase Bubblegum Family Member 1
GK2: Glycerol Kinase 2
CPT2: Carnitine Palmitoyltransferase 2
GPX4: Glutathione Peroxidase 4
NRF2: NFE2 Like BZIP Transcription Factor 2
NCOA4: Nuclear Receptor Coactivator 4
KEAP1: Kelch Like ECH Associated Protein 1
