# Supplementary material for: Identification and validation of a fatty acid metabolism-related lncRNA signature as a predictor for prognosis and immunotherapy in patients with liver cancer
Source: BMC Cancer. 2022 Oct 4;22:1037. doi: 10.1186/s12885-022-10122-4 (PMC9531484; doi:10.1186/s12885-022-10122-4)
Supplement: Supplementary file 6 — Additional file6: Supplementary figure 1. The expression of immune checkpoint genes between FA metabolism-related lncRNA subgroups. (A, B) The boxplots display the dysregulation of immune checkpoint genes in the TCGA and GSE76427 cohorts. [file 12885_2022_10122_MOESM6_ESM.docx]

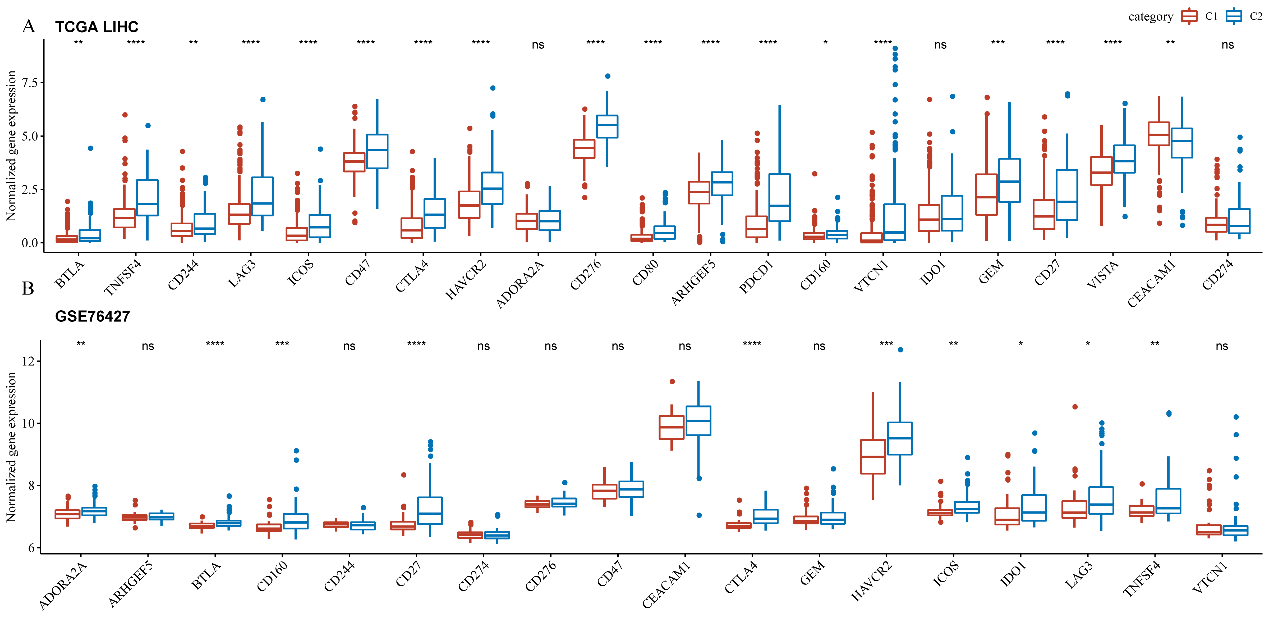


**Suppl Figure 1.** The expression of immune checkpoint genes between FA metabolism-related lncRNA subgroups. (**A, B)** The boxplots display the dysregulation of immune checkpoint genes in the TCGA and GSE76427 cohorts.
