# Supplementary material for: Identification and validation of a fatty acid metabolism-related lncRNA signature as a predictor for prognosis and immunotherapy in patients with liver cancer
Source: BMC Cancer. 2022 Oct 4;22:1037. doi: 10.1186/s12885-022-10122-4 (PMC9531484; doi:10.1186/s12885-022-10122-4)
Supplement: Supplementary file 7 — Additional file7: Supplementary figure 2. Original gels for all western blots in Figure 9 Original gel image measuring immunopositivity against ferroptosis markers in HCC cells. ACTIN was used as loading control. Bands used in the manuscript have been boxed in red. [file 12885_2022_10122_MOESM7_ESM.docx]

**Supplementary figure 2：Original gels for all western blots in Figure 9**


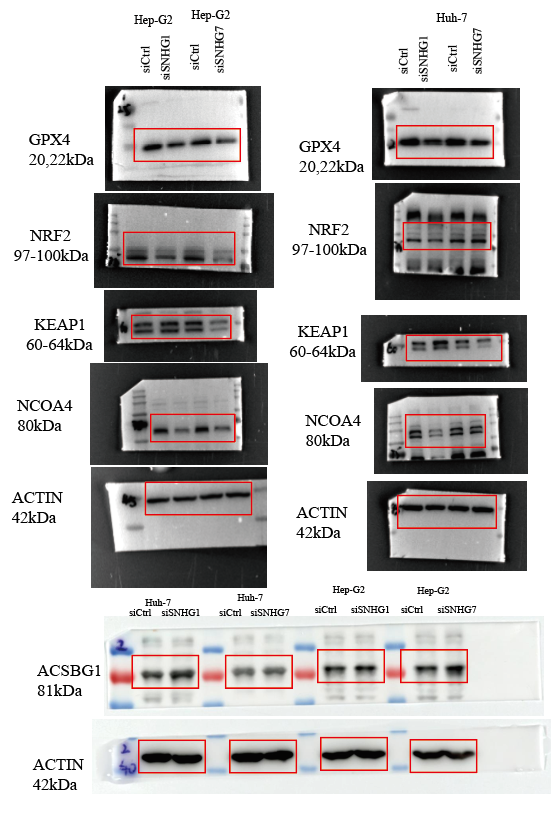


**Figure legend**: Original gel image measuring immunopositivity against ferroptosis markers in HCC cells. ACTIN was used as loading control. Bands used in the manuscript have been boxed in red.
