# Supplementary material for: Identification and validation of a fatty acid metabolism-related lncRNA signature as a predictor for prognosis and immunotherapy in patients with liver cancer
Source: BMC Cancer. 2022 Oct 4;22:1037. doi: 10.1186/s12885-022-10122-4 (PMC9531484; doi:10.1186/s12885-022-10122-4)

**Original images for Figure 9**

GPX4 HepG2 siCtrl siSNHG1 siCtrl siSNHG7 NRF2 HepG2 siCtrl siSNHG1 siCtrl siSNHG7


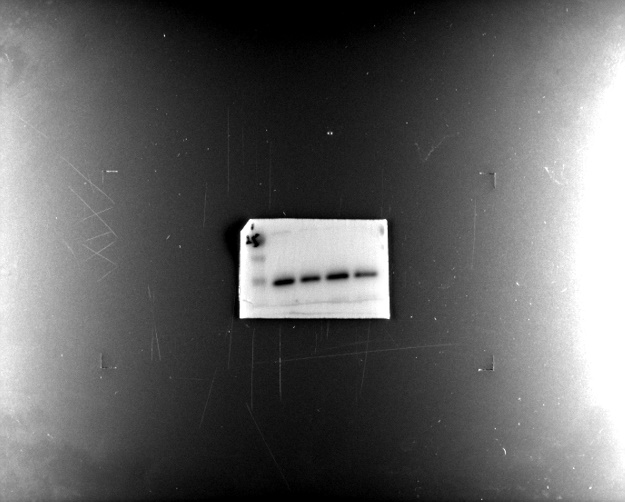

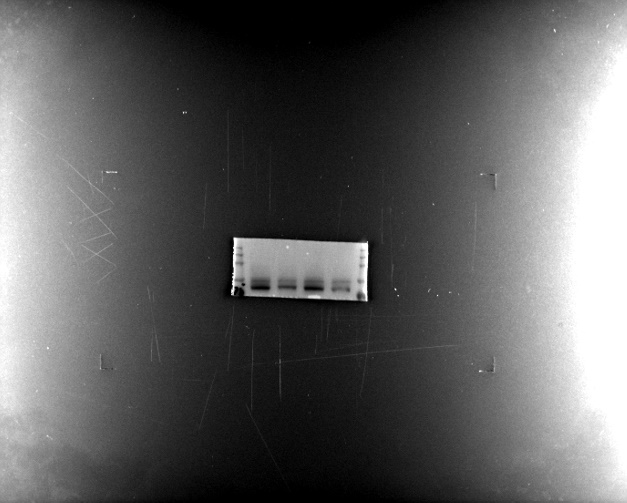


KEAP1 HepG2 siCtrl siSNHG1 siCtrl siSNHG7 NCOA4 HepG2 siCtrl siSNHG1 siCtrl siSNHG7
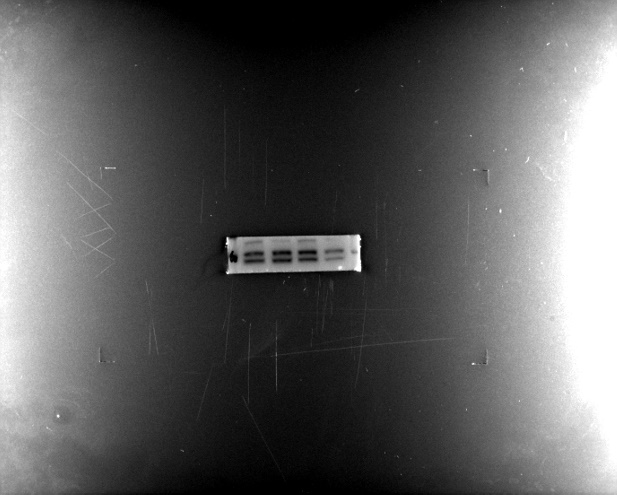

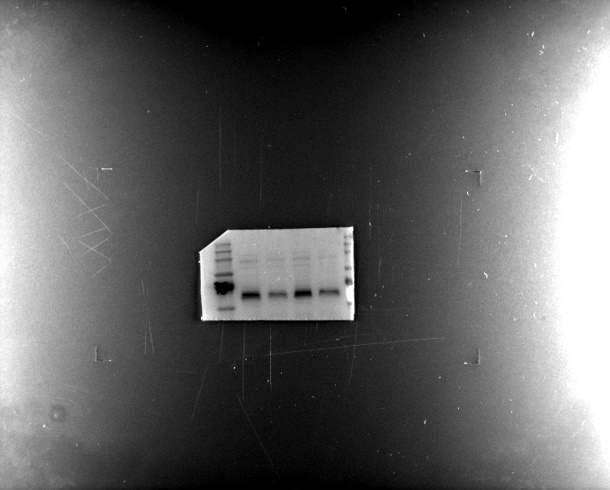


ACTIN HepG2 siCtrl siSNHG1 siCtrl siSNHG7


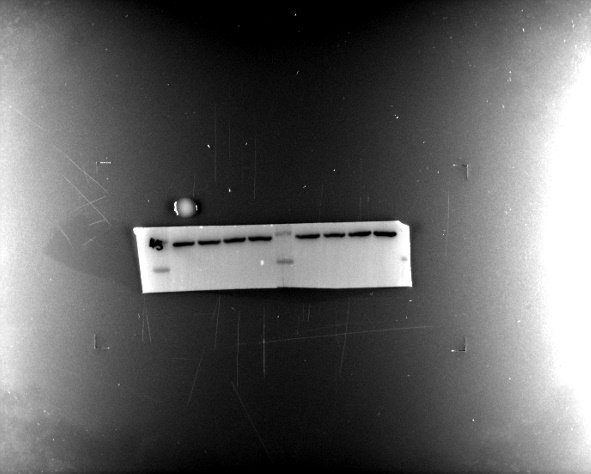
\

GPX4 Huh7 siCtrl siSNHG1 siCtrl siSNHG7 NRF2 Huh7 siCtrl siSNHG1 siCtrl siSNHG7


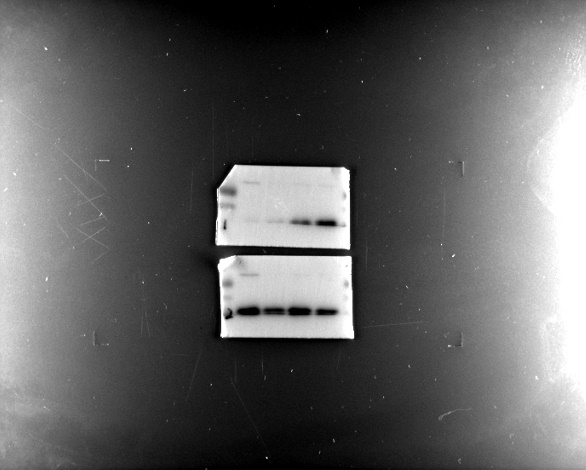

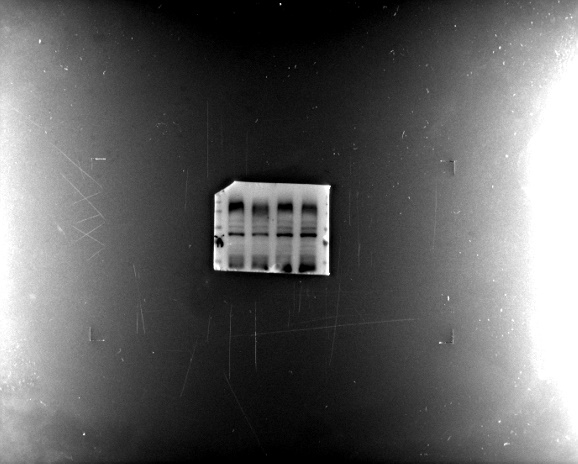


KEAP1 Huh7 siCtrl siSNHG1 siCtrl siSNHG7 NOCA4 Huh7 siCtrl siSNHG1 siCtrl siSNHG7


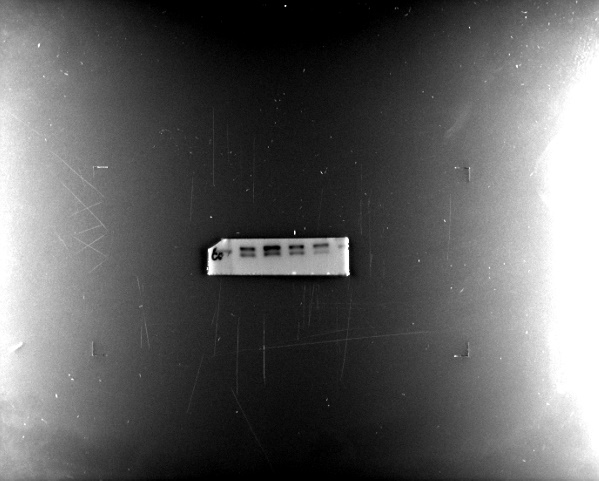

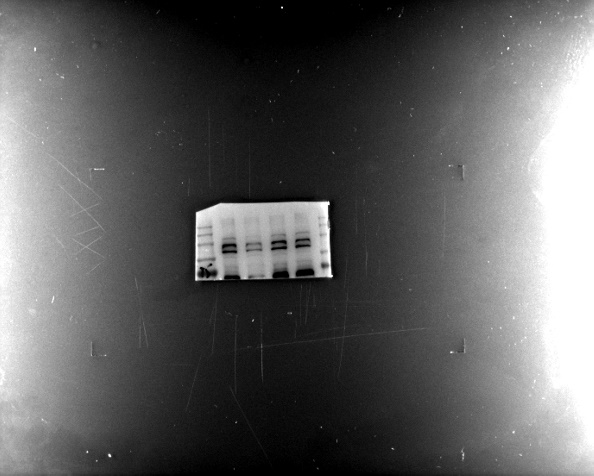


ACTIN Huh7 siCtrl siSNHG1 siCtrl siSNHG7


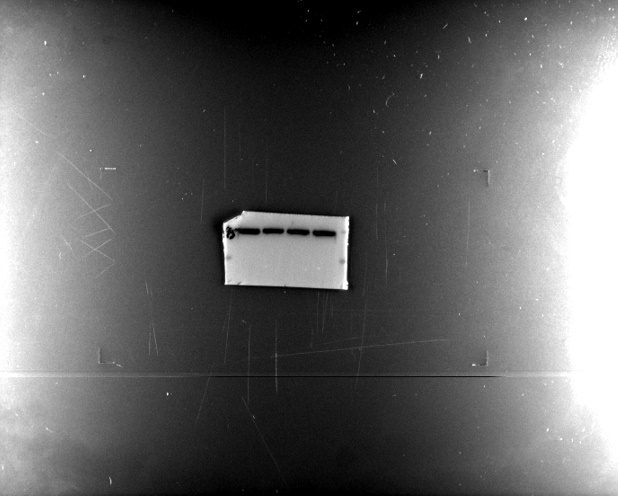


ACSBG1

Huh7(siCtrl siSNHG1 siCtrl siSNHG7) HepG2(siCtrl siSNHG1 siCtrl siSNHG7)


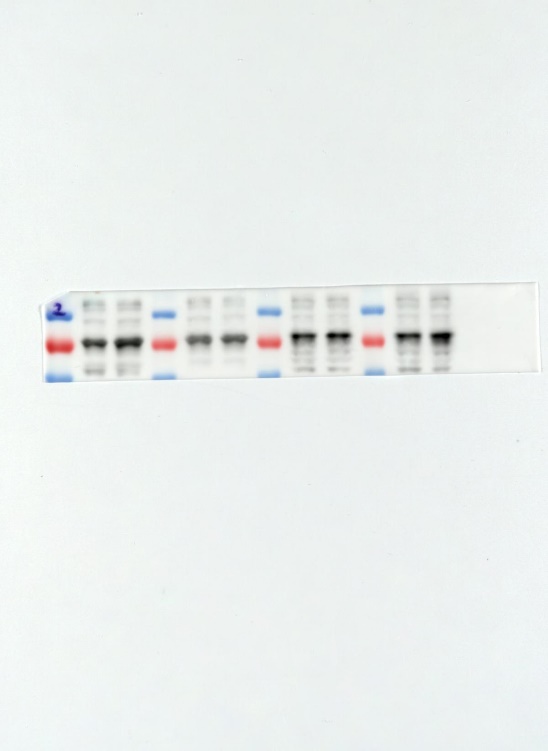


ACTIN

Huh7(siCtrl siSNHG1 siCtrl siSNHG7) HepG2(siCtrl siSNHG1 siCtrl siSNHG7)


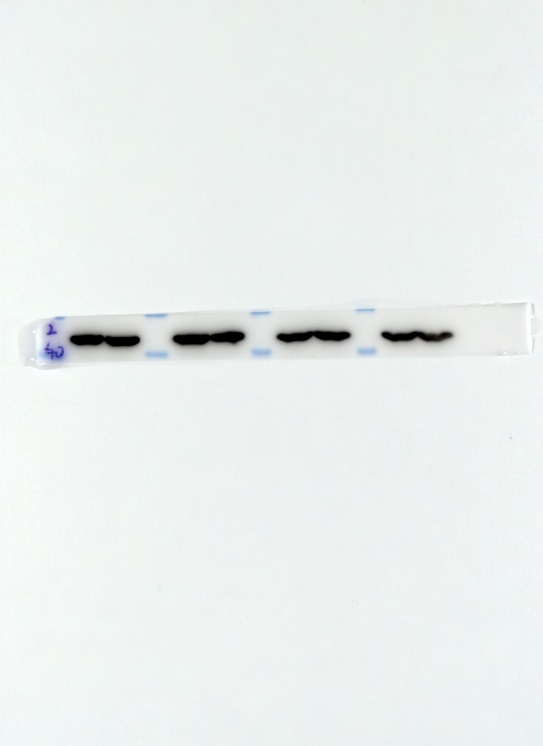

Supplement: Supplementary file 8 — Additional file8: Supplementary figure 3. Original gels images for all western blots in Figure 9. [file 12885_2022_10122_MOESM8_ESM.docx]
