# Supplementary material for: Identification and validation of a fatty acid metabolism-related lncRNA signature as a predictor for prognosis and immunotherapy in patients with liver cancer
Source: BMC Cancer. 2022 Oct 4;22:1037. doi: 10.1186/s12885-022-10122-4 (PMC9531484; doi:10.1186/s12885-022-10122-4)
Supplement: Supplementary file 9 — Additional file9: Supplementary Table 1. Sequences of Primers used for qRT-PCR. [file 12885_2022_10122_MOESM9_ESM.docx]

**Table S1 Sequences of Primers used for qRT-PCR.**

| **SNHG1** |  |
| --- | --- |
| Forward | 5’- CCCGTCATGAGCCTTTAGGT -3’ |
| Reverse | 5’- AGCAACACAAATTGACAGCCA -3’ |
| **SNHG7** |  |
| Forward | 5’- GTGACTTCGCCTGTGATGGA-3’ |
| Reverse | 5’- GGCCTCTATCTGTACCTTTATTCC-3’ |
| **HAVCR2** |  |
| Forward | GGTCATCAAACCAGCCAAGGT |
| Reverse | CATGTCCCCTGGTGGTAAGC |
| **ICOS** |  |
| Forward | TTGAACACTGAACGCGAGGA |
| Reverse | GCAGAACCATTGATTTCTCCTGT |
| **LAG3** |  |
| Forward | TCACAGAGCTGTCTAGCCCA |
| Reverse | TCTTGGTCGCCACTGTCTTC |
| **PDCD1** |  |
| Forward | CAGTTCCAAACCCTGGTGGT |
| Reverse | GGCTCCTATTGTCCCTCGTG |
| **FOXO1** |  |
| Forward | GAGGGTTAGTGAGCAGGTTACA |
| Reverse | TGCTGCCAAGTCTGACGAAA |
| **HNF1A** |  |
| Forward | GCCTCACGCCCACCAAG |
| Reverse | CTGCTGGAGGACACTGTGG |
| **PPARA** |  |
| Forward | CCTGTCTGCTCTGTGGACTC |
| Reverse | GACCAGATGGTGCTGGTTGT |
| **PPARG** |  |
| Forward | GGTGACCAGAAGCCTGCAT |
| Reverse | AGGAGTGGGAGTGGTCTTCC |
| **CEBPA** |  |
| Forward | AAGCACGATCAGTCCATCCC |
| Reverse | GGCACAGAGGCCAGATACAA |
| **ONECUT1** |  |
| Forward | CAGATGTCCAGCGTCGAACT |
| Reverse | GCCACTTGTCCAGACTCCTC |
| **CREB1** |  |
| Forward | TTAGTGCCCAGCAACCAAGT |
| Reverse | AATGTATGGTTTGAGTGGAAAAGAT |
| **FOXA1** |  |
| Forward | GAAGACCGGCCAGCTAGAGG |
| Reverse | TGTTGATGGAGAACGGGTGG |
| **ZBTB7A** |  |
| Forward | ATGCACCCCCTCGCAATAAA |
| Reverse | AGGGTTTAGTGCAATCCCCG |
| **CEBPB** |  |
| Forward | ACGCAACCCACGTGTAACT |
| Reverse | CCCCAAAAGGCTTTGTAACCATT |
| **E2F4** |  |
| Forward | TTGATCCCACACGAGAGTGC |
| Reverse | GGGTGGAGAAAGACGAAGCA |
| **SMAD3** |  |
| Forward | AGCGCACTGACCATAAGAGC |
| Reverse | AGTGTGGGTTTCCATGCAGT |
