# Supplementary material for: Identification and validation of a fatty acid metabolism-related lncRNA signature as a predictor for prognosis and immunotherapy in patients with liver cancer
Source: BMC Cancer. 2022 Oct 4;22:1037. doi: 10.1186/s12885-022-10122-4 (PMC9531484; doi:10.1186/s12885-022-10122-4)
Supplement: Supplementary file 10 — Additional file10: Supplementary Table 2. Sequences of siRNA and smart silencer used for lncRNA knockdown. [file 12885_2022_10122_MOESM10_ESM.docx]

**Table S2 Sequences of siRNA and smart silencer used for lncRNA knockdown.**

| **Smart silencer for SNHG1** | Target sequence |
| --- | --- |
|  | CCAGCATCTCATAATCTAT |
|  | GTGAAGGAATGGGACAAGAC |
|  | CCCTTGAGGACTGGCTGTCA |
|  | AGCTGAGAGGTACTACTAAC |
|  | GAGGACATCAGAAGGTGAA |
|  | GCCAGCACCTTCTCTCTAA |
| **SiRNA for SNHG7** |  |
|  | CCAGUUCUCGAGCGCCUCA |
