# Supplementary material for: Identification and validation of a fatty acid metabolism-related lncRNA signature as a predictor for prognosis and immunotherapy in patients with liver cancer
Source: BMC Cancer. 2022 Oct 4;22:1037. doi: 10.1186/s12885-022-10122-4 (PMC9531484; doi:10.1186/s12885-022-10122-4)
Supplement: Supplementary file 11 — Additional file11: Supplementary Table 3. List of Primary Antibodies used in the study. [file 12885_2022_10122_MOESM11_ESM.docx]

**Table S3 List of Primary Antibodies used in the study.**

| Antibody | Applications | Company |
| --- | --- | --- |
| GPX4 | WB | CST (52455) |
| ACTIN | WB | CST (3700) |
| NRF2 | WB | CST (12721) |
| KEAP1 | WB | CST (8047) |
| NOCA4 | WB | CST (66849) |
| ACSBG1 | WB | Proteintech (16077-1-AP) |

**Abbreviations:** WB, western blot
